# Long-term clinical outcome for patients poisoned by the fungal nephrotoxin orellanine

**DOI:** 10.1186/s12882-017-0533-6

**Published:** 2017-04-03

**Authors:** Heidi Hedman, Johan Holmdahl, Johan Mölne, Kerstin Ebefors, Börje Haraldsson, Jenny Nyström

**Affiliations:** 1 Department of Molecular and Clinical Medicine, Institute of Medicine, the Sahlgrenska Academy, University of Gothenburg, Gothenburg, Sweden; 2 Department of Physiology, Institute of Neuroscience and Physiology, the Sahlgrenska Academy, University of Gothenburg, PO Box 432, SE-40530 Gothenburg, Sweden; 3 Department of Nephrology, Institute of Medicine, The Sahlgrenska Academy, University of Gothenburg, Gothenburg, Sweden; 4 Department of Pathology, Institute of Biomedicine, The Sahlgrenska Academy, University of Gothenburg, Gothenburg, Sweden

## Abstract

**Background:**

Accidental intake of mushrooms of the *Cortinarius* species (*deadly webcap)* may cause irreversible renal damage and the need for dialysis or transplantation. The species is found in forests of Northern Europe, Scandinavia and North America and may be mistaken for other edible mushrooms. The highly selective nephrotoxic compound of the mushroom is called orellanine. Very little is known about the long-term effects of the nephrotoxin.

**Methods:**

We identified patients who ingested *deadly webcap* in the period of 1979 to 2012. Informed consent and medical records were obtained for 28 of the 39 cases that occurred during the 34-year period. A case control group was also studied based on sex, age and initiation of dialysis or transplantation.

**Results:**

The average age at time of the accidental intake was 40 ± 3 (*n* = 28) years. 64% of patients were male, and 22 of 28 patients developed acute kidney injury requiring dialysis. Serum creatinine peaked at 1 329 ± 133 μmol/l, and serum urea was 31 ± 3.5 mmol/l. No signs of acute damage were present in any other organ. The average time of follow-up was 16.9 ± 2.1 years (1.24–34.3 years, *n* = 28). 15 patients were transplanted and 3 also had a second graft. At follow-up, 23 patients were alive, and five had died at ages of 67 ± 5 (range 54–84). The outcome was similar in the case control group with 6 deaths in 20 patients.

**Conclusion:**

We conclude that the long-term prognosis for patients poisoned by deadly webcap who lost their renal function is not different compared to other patients in active uremic care.

## Background

**Fig. 1:**
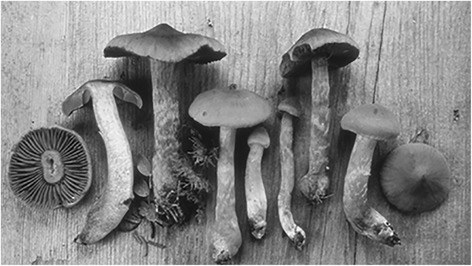
Specimens of the deadly webcap or *Cortinarius rubellus* (synonymous with *Cortinarius speciosissimus*), younger and older mushrooms with slightly different appearance. Picture by Hans Marklund

The mushroom known as the deadly webcap or *Cortinarius rubellus* (synonymous with *Cortinarius speciosissimus*) is occasionally mistaken for eatable chanterelles [1–5], Fig. 1. The habitats are unevenly distributed in Europe and North America and reports of poisoning episodes have been made from forestry areas throughout these regions. One to three of the deadly webcaps is considered enough to cause severe renal failure. There are usually no acute symptoms and the patient will seek medical advice 3 days to a week later with the symptomatology of uraemia caused by an acute kidney injury. The main symptoms of the uraemia at this stage are nausea and fatigue. Edema is less common due to an initial polyuric phase. This is in turn due to the tubulo-interstitial nephritis caused by damage to the tubular cells. In case of more severe poisoning, the patient becomes anuric after around 1 week [4].

The deadly webcap contains a highly selective nephrotoxin, orellanine, first described by Grzymala [6]. Orellanine (or [2, 2′-bipyridine]-3, 3′,4, 4′-tetrol-1, 1′-dioxide) is present in the mushroom in its basic form as a di-glycoside [7, 8]. The mechanism of action is that orellanine generates oxygen radicals [9–11] and simultaneously shuts down the oxidative defence, by down-regulating most anti-oxidative enzymes [9]. In rodents, the highly kidney-specific nature of orellanine has been verified. Thus, no acute symptoms have been observed at doses 1–2 orders of magnitude higher than the dose required to elicit acute renal failure [12, 13].

There are several case reports of the acute intoxication [1, 2, 14–18]. Due to the lag-phase between intake and symptoms, there are no signs of toxins in blood [19]. Suggested therapies during the years have been hemodialysis, hemoperfusion, corticosteroids and anti-oxidant therapies with highly varying and inconclusive results [4, 20, 21]. Regarding long-term effects of the intoxication, there are only a few reports following transplantation [17, 22]. There are no long-term follow up on patients after the initial acute kidney injury phase. Therefore, we tried to identify all cases of deadly webcap intoxication during the last three decades in the most affected (South-Western) region of Sweden and compared their outcome with a case-control group of patients. Our hypothesis was that the poison might have long-term effects or increase patient morbidity and mortality. Hence, a long time follow up of a patient cohort would be of interest.

## Methods

### Study population

We included all patients accidentally poisoned by intake of the mushroom deadly webcap (or *Cortinarius rubellus*, also known as *Cortinarius speciosissimus*) admitted to any of the hospitals in the Western part of Sweden during the period of January 1979 to December 2012. The total number of patients was 39. In this report, 28 patients were included based on informed consent and available medical records, see Table 1 for demographic data.

**Table 1:** Demographic data of the Cortinarius intoxicated patient cohort

						Cont on dialysis		GFR	GFR Year	Age at intox	Admission Year				Crea	Alat	Alp	Bil	Urea	Asat	Cancer
No.	Biopsy (Y/N)	† Year	1st trpl Year	2nd trpl Year	Initial dialysis		CKD5					BP	Hb	Alb	Inital peak values first w. after intoxication						after intox
1^a^	N				N	N	N			47	1979	130/70	116	39	889	0,22	3,6	5	9		N
2^a^	N				N	N	N			24	1979	140/80	149	P 71	400	0,19	2,9	9		0,33	
3	N		1980		Y	N	Y	35	2011	44	1979	185/100	124		1550	*0,3*	*3*	*6*	37	*0,3*	Y^b^
4	N		1986		Y	N	Y	55	2012	60	1985	170/110	95	P 71	542	0,54	4,1	7,5	23	0,52	Y^c^
5	Y				Y	N	N			26	1985	120/80	86		1270	*0,3*	*3*	*7*	17	*0,3*	N
6^a^	N		1987	1999	Y	N	Y	38	2004	21	1985										
7	Y		1988	2003	Y	N	Y	107	2009	14	1987	130/80	101		1800				24		N
8	N	2012			N	N	N			40	1987	130/80	144	P 67	126	0,7	2,2	11		0,47	
9^a^	N				Y	Y	Y			26	1990										
10	N				Y	Y	Y			16	1990										
11	N		1992	2004	Y	N	Y			39	1991	130/70	123	32	1333	0,47	3	7,4	45	0,23	N
12	N		1992		Y	N	Y			41	1991	130/80	119	32	1074	0,19	3,3	7,9	34	0,17	N
13	N		1992		Y	N	Y			21	1991	135/80	125	45	1458	0,14	2,7	7,5	42	0,24	Y^d^
14	N	1999	1983		Y	N	Y			41	1979	160/90	143	41	1630				46		
15	N	2000			Y	Y	Y			68	1984	180/95	125	P 72	970	*0,3*	*3*	*7*		*0,3*	
16	N		2000		Y	N	Y	28	2003	28	1999										
17	N		2001		Y	N	Y	52	2002	27	1999										
18	N		2002		Y	N	Y	44	2012	36	2001				1100	*0,3*	*3*	*7*		*0,3*	N
19	N	1995			Y	Y	Y	40		60	1984	180/100	111	P 69	970	0,26	2,2	14	10	0,31	
20	Y	2007			Y	Y	Y			46	1999										
21	N		2013		Y	N	Y			48	2007	129/71			1052	0,28	1,4	6,4	27,6	0,3	N
22	N		2009		Y	N	Y	40	2009	52	2007	174/97									N
23^a^	N				N	N	N			25	2010	160/104	115		243	0,45	1,1	6			
24	Y		2012		Y	N	Y	11	2012	57	2010	125/87	127	37	2809	0,12	1	7,9			N
25^a^	N				Y	Y	Y			61	2011	145/80	98	23	1300				35		
26	N				Y	Y	Y			39	2011	150/80	137	30	1081	0,22	0,62	5		0,47	N
27^a^	N				N	N	N			48	2011	140/80	152	34	710	0,6	1,3	6,9	25,2	0,72	N
28^a^	Y				N	N	N			55	2012	125/80	152	33	473	0,51	1,5	8,2			N

Patient data.^a^ = no matched control available, F: female, M: male, TRPL: kidney transplantation, CKD: Chronic kidney disease, GFR: measured glomerular filtration rate, BP: blood pressure, Hb: hemoglobin value in g/L, Alb: serum albumin (in some cases protein was measured: P) g/L, Crea: creatinine in umol/L, Alat / Asat / ALP, values measured in μCat/L bilirubin (bil) measured in μmol/L. Liver values reported as normal was estimated *(shown in italics). *Y^b^: Oral cancer, Y^c^: Skin cancer, Y^d^: Hodgkins Lymphoma

### Case control population

For each patient included in the study population that developed CKD5 (chronic kidney disease stage 5), we randomly included another patient as case control from the dialysis unit and another from the transplantation centre at Sahlgrenska University Hospital. Patients with diabetes or other systemic disease were excluded. The case controls were matched in age, sex, comorbidity and the year of the initiation of dialysis or transplantation. Such case control subjects could be found for all study patients in the dialysis group. However, three case controls are missing during certain years for the transplantation group due to large age differences. Most of the case control patients included had interstitial nephritis (75%), glomerulonephritis, or polycystic kidney disease, see Table 3 for demographic data.

### Kidney morphology

Kidney biopsies were taken using gauge 16 needles, fixed in buffered paraformaldehyde, embedded in paraffin, sectioned and stained with standard dyes (haematoxylin-eosin, trichrome, silver and periodic acid-Schiff) and evaluated by a renal pathologist.

### Statistical analysis

*Results are presented as mean ± SEM (standard error of the mean). Differences between groups were calculated using the Gehan-Breslow-Wilcoxon test.*

## Results

### Study population

Of the 39 patients identified, we were able to include 28 patients in the study. This was mainly due to difficulties to be able to get in contact and obtain an informed consent form from all patients.

Of the 28 patients, 22 required dialysis initially, and 6 did not, Table 1. After 1 month, 21 patients had CKD5 requiring dialysis or transplantation and 7 patients had CKD stage 3. Thus, there was a 4.5% chance for a patient initially requiring dialysis to regain renal function during the first month after the intoxication (1/22). Of the 7 patients that were dialysis-free 1 month after the intoxication, all regained most of their renal function within the first year reaching CKD stage 1 or 2.

For the total study population of 28 patients, the average time of follow-up was 16.9 ± 2.1 years (minimum 1.24 and maximum 34.3 years). 23 patients were alive at the time of follow up, but five had died at ages of 67.0 ± 5.1 years. Over the long observation period, three patients with end stage renal disease (ESRD) and hemodialysis died at the age of 70 ± 9 years after 11.9 ± 2.5 years on dialysis.

One patient who never required dialysis died 24 years after the intoxication at the age of 65. Another patient died 19.1 years after the intoxication and 16 years after a successful kidney transplant at the age of 61. Fourteen patients were transplanted and of these three had a second kidney graft 12–15 years after the first one. The average age at the time of the accidental intake of poisonous mushrooms was 40 ± 3 years (*n* = 28), and 64% of patients were male. In the study group, 3 of the 16 transplanted patients developed cancer in the form of lymphoma, oral or skin cancer. Information of the specific cancer diagnosis was not available for the case control group.

#### Acute effects

The serum creatinine of the patients requiring dialysis peaked at 1 329 ± 133 μmol/l (*n* = 15) and serum urea reached 31 ± 3.5 mmol/l (*n* = 11). The peak creatinine and urea values were markedly lower in patients that did not require dialysis, Table 1. Laboratory tests did not show signs of damage in any other organ apart from the kidney. Thus, the aspartate amino transferase (ASAT), alanine amino transferase (ALAT), alkaline phosphatase (ALP) and bilirubin values were all within their normal limits in the patients analyzed; 0.35 ± 0,04 μCat/l, 0.34 ± 0.04 μCat/l, 2.38 ± 0.04 μCat/l, and 7.59 ± 0.5 μmol/l respectively (n_ALAT_ = 14, n_ASAT, ALP, bilirubin_ = 18).

#### Patients with non-dialysis dependent kidney injury

Six of the patients that were orellanine intoxicated developed a less severe kidney injury and never required dialysis. On average their serum creatinine peaked at 474 ± 117 μmol/l (*n* = 3) during the first week. Initially, two patients were treated with three sessions each of hemoperfusion with charcoal, which in 1979 was suggested as therapy. One patient initially required dialysis and had a serum creatinine of 1270 μmol/l, but regained part of her renal function within the first month, and had a glomerular filtration rate of 87 ml/min 1 year later determined by ^51^Cr-EDTA clearance. The 7 patients who did not develop CKD5 were followed over a time-period of 18.3 ± 5.8 years, range 1.2–34.3 years.

#### Patients with dialysis-dependent chronic kidney disease stage 5

One month after the intoxication, 21 of 22 patients still required dialysis. 14 of them were transplanted, 6 received a kidney graft from a living donor and 8 from a dead donor. Before transplantation, the patients had been on dialysis for 19 ± 4 months (range 7–64 months). Three renal grafts lost their function after 11, 12 and 15 years and a second kidney transplantation was performed. Those patients now have been living with their 2nd grafts for 10, 11 and 15 years, Table 1.

#### Kidney morphology

**Fig. 2:**
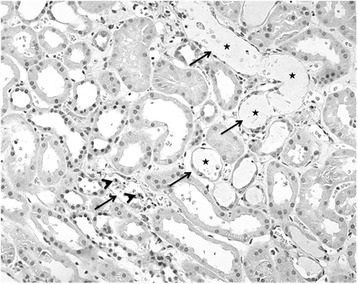
Light microscopy from a Cortinarius intoxicated patient showing proximal tubular damage (arrows) in some tubules. There are focal apoptotic bodies (arrowheads), denudation and tubular dilatation (★). Haematoxylin-eosin stain

Kidney biopsies were obtained from 5/28 patients. The biopsy material from one patient could not be retrieved and thus material was available from 4 patients. According to clinical data biopsies were obtained 10–21 days after fungal ingestion. All biopsies showed a similar pattern dominated by acute tubular necrosis (ATN), Fig. 2. Two patients (no 5 and 28) showed less severe damage with focal involvement, while the other two biopsies (no 20 and 24) showed a diffuse involvement with generalized inflammation and tubular damage. These biopsies also showed tubular dilatation, tubular cellular atrophy and degeneration, Table 2. All biopsies contained some apoptotic bodies, interstitial edema and focal inflammation but no eosinophilia. Fibrosis was not a prominent finding.

**Table 2:** Renal morphology of biopsies from patients with Cortinarius poisoning

Patient No.	Time after ingestion	Tubular damage: Necrosis, apoptosis, degeneration, dilatation, involvement		Edema	Inflammation	Fibrosis
5	2v	0 + 0 0	focal	minimal	minimal	minimal
7	--	---		-	-	-
20	3v	+ + + +	diffuse	severe	prominent	minimal
24	>10d	0 + + +	diffuse	prominent	slight	slight
28	10d	0 + 0 0	focal	focal	minimal	minimal

### Case control population

**Fig. 3:**
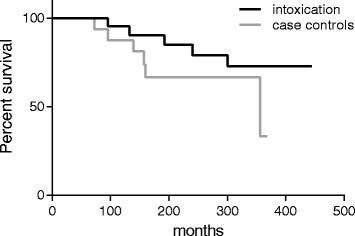
Kaplan Mayer survival curve over the two groups. No statistical differences were seen between the study group (intoxicated patients) and the control group over time

The case control patients were age and sex matched, and they started dialysis or were transplanted in the same year as the patients in the study population, Table 3. No acceptable case control could be found for three of the 16 transplanted study patients since the candidates were either too old or had systemic disease. The distribution of kidney disease within the population is displayed in Table 3. In total, 20 patients were matched, 7 of the patients were on dialysis and the remaining 13 patients were transplanted, one of them twice. Of the 20 patients, 6 died at a mean age of 67.5 ± 4.7 years after 16.9 ± 6.6 years on dialysis (*n* = 3, at a mean age of 68 years) and 10.3 ± 2.2 years after transplantation (*n* = 3, at a mean age of 66 year), Fig. 3. In the case control population, one patient died of cancer and three other patients developed tumours during the observation period.

**Table 3:** The table describes the demographic data available for the matched control cohort^a^second transplant in 2003

A. Matched control patients who had hemodialysis								
No#:	Born year	Transplant 1	Diagnosis	Follow up M	Follow up Y	Death Y	CoD	Cancer
1	1935	1980	PCK	356	30	2010	Peritonitis	Y
2	1925	1986	Amylioidosis	95	8	1994	Unknown	Y
3	1965	2002	Crescentic GN	141	12			N
4	1953	2012	IgAN	20	2			N
5	1958	2012	PCK	22	2			N
6	1955	2009	PCK	59	5			N
7	1942	1983	Pyelonephritis	368	31			
8	1972	1988^a^	PCK	307	26			N
9	1970	2000	Interstitial Nephritis	156	13			
10	1973	2001	IgAN	194	16			
11	1952	1992	IgAN	157	13	2005	Epilepsy	N
12	1951	1992	Interstitial nephritis	263	22			N
13	1971	1992	CGN	260	22			
B. Matched control patients who had a renal transplantation								
No#:	Born year	HD start	Diagnosis	Follow up M	Follow up Y	Death Y	CoD	Cancer
14	1925	1984	CVD	160	13	1997	Cancer	Y
15	1916	1984	Heredetary nephropaty	139	12	1996	Sepsis	
16	1962	1985	Glomerulonephritis	343	29			Y
17	1942	1987	CVD	72	6	1993	CVD	
18	1965	1990	Glomerulonephritis	284	24			
19	1952	1999	Glomerulonephritis	178	15			
20	1973	2011	Interstitial nephritis	29	2			N

^a^Second transplant in 2003, *M*: months, *Y*: year, PCK:polycystic kidney disease, Crescentic *GN*: crescentic glomerulonephritis, *IgAN*: IgA nephritis, *CGN*: Chronic glomerulonephritis, *CoD*: cause of death, *CVD*: cardiovascular disease

The cohort was matched based on age, sex and A. initiation of hemodialysis or B. renal transplantation

### Comparisons between study and control populations

Of the 21 patients with ESRD in the study group, 20 were matched; 7 in dialysis and 13 that were transplanted. Cancer was not more frequent in the study group compared to control group. There were 3 cases of cancer in the study population of 21 patients (14%), of which all 3 were transplanted. In the control group 3 out of 20 (15%) were diagnosed with cancer, two of which were transplanted and one which remained in dialysis. In total, 4 patients of 21 with ESRD died in the study group compared to 6 in the control group, see Fig. 3. There was no statistical difference between survivals in the study group compared to the case control group.

## Discussion

The present study was done to reveal if patients that accidentally ingested deadly webcap (Cortinarius rubellus) suffered from long-term increases in morbidity or mortality. Indeed, 75% of the 28 patients developed CKD5, and 70% of them were transplanted. However, we did not find evidence of any other damage apart from the severe kidney injury. It should be noted that this is still a small patient group and of 39 cases we were only able to investigate 28 cases. Possible confounders could be the limited size of the study or that bias was introduced by co-morbidities or other medication. However, we do not anticipate that this has affected the outcome of the study.

The follow up period after the accidental intake of poisonous webcap is quite long, 16.9 years, with a range of up to 34.3 years. As expected, the long-term survival was excellent after transplantation as reflected by the current follow up period of 19.0 ± 2.5 years for the 14 patients in that group. The result is in accordance with previous reports over shorter periods of follow up [17, 22]. Regarding dialysis, it is well known that the mortality is high for all age groups [23]. Naturally, the survival on dialysis will depend, not only on the dialysis practice, but also on the underlying disease and on comorbidities [23–25]. The outcome was quite good for the study population on dialysis, which so far has been followed for 12.4 ± 3.4 years. Some patients have died over the three and a half decades of follow up. However, the patients on dialysis died after more than 12 years of treatment with haemodialysis at a mean age of 68. A similar picture was found in the case control population with follow up periods for transplanted patients of 15.2 ± 2.8 years and of patients on dialysis for 14.4 ± 3.5 years. In the case control population there were three deaths in each of the two ESRD groups, with transplanted patients being 66 years at the time of death, and patients on dialysis being 68. We found no evidence of increased risk for malignancy, endocrinological disorders or other morbidity in the study population.

## Conclusion

We conclude that the long-term outcome is equally good for patients that have lost their renal function due to accidental intake of deadly webcap compared to other reasons for the uraemia. This was true both for transplanted patients and patients treated with dialysis.

## Abbreviations

^51^Cr-EDTA: 51 Chromium-Ethylenediaminetetraacetic acid
ALAT: Alanine amino transferase
ALP: Alkaline phosphatase
ASAT: Aspartate amino transferase
ATN: Acute tubular necrosis
CKD5: Chronic kidney disease stage 5
ESRD: End stage renal disease
SEM: Standard error of the mean
